# Supplementary material for: Addressing mechanism bias in model-based impact forecasts of new tuberculosis vaccines
Source: Nat Commun. 2023 Sep 1;14:5312. doi: 10.1038/s41467-023-40976-6 (PMC10474143; doi:10.1038/s41467-023-40976-6)
Supplement: Supplementary file 3 — Reporting Summary [file 41467_2023_40976_MOESM3_ESM.pdf]

nature portfolio

Corresponding author(s): Joaquín Sanz

Last updated by author(s): Jul 10, 2023

Reporting Summary

Nature Portfolio wishes to improve the reproducibility of the work that we publish. This form provides structure for consistency and transparency in reporting. For further information on Nature Portfolio policies, see our Editorial Policies and the Editorial Policy Checklist.

Statistics

For all statistical analyses, confirm that the following items are present in the figure legend, table legend, main text, or Methods section.

n/a	Confirmed	
☐	☒	The exact sample size (*n*) for each experimental group/condition, given as a discrete number and unit of measurement
☐	☒	A statement on whether measurements were taken from distinct samples or whether the same sample was measured repeatedly
☐	☒	The statistical test(s) used AND whether they are one- or two-sided<br>*Only common tests should be described solely by name; describe more complex techniques in the Methods section.*
☒	☐	A description of all covariates tested
☐	☒	A description of any assumptions or corrections, such as tests of normality and adjustment for multiple comparisons
☐	☒	A full description of the statistical parameters including central tendency (e.g. means) or other basic estimates (e.g. regression coefficient) AND variation (e.g. standard deviation) or associated estimates of uncertainty (e.g. confidence intervals)
☐	☒	For null hypothesis testing, the test statistic (e.g. *F*, *t*, *r*) with confidence intervals, effect sizes, degrees of freedom and *P* value noted<br>*Give P values as exact values whenever suitable.*
☐	☒	For Bayesian analysis, information on the choice of priors and Markov chain Monte Carlo settings
☒	☐	For hierarchical and complex designs, identification of the appropriate level for tests and full reporting of outcomes
☒	☐	Estimates of effect sizes (e.g. Cohen's *d*, Pearson's *r*), indicating how they were calculated

Our web collection on statistics for biologists contains articles on many of the points above.

Software and code

Policy information about availability of computer code

Data collection	No software was used for data collection.
Data analysis	Code with the implementation of the novel methods introduced in this study is available at GitHub (https://github.com/MarioTovarCalonge/Bayesian_Framework_TB_Vaccines) and at Zenodo (https://zenodo.org/badge/latestdoi/596053638). Those codes include algorithms written in C language (in-house Gillepie algorithm-based implementation of clinical trial simulations), and in R (tested in version 3.6.3), with dependences, at different stages, to the R packages: fanplot(v4.0.0), ggplot2(v3.4.0), gridExtra(v2.3), kdensity(v1.1.0), KernSmooth(v2.23-20), viridis(v0.6.2), truncnorm(v1.0-8), minpack.lm(v1.2-2), nlsr(v2019.9.7), iterators(v1.0.14), foreach(v1.5.2), doParallel(v1.0.17), and dplyr (v1.0.10).

For manuscripts utilizing custom algorithms or software that are central to the research but not yet described in published literature, software must be made available to editors and reviewers. We strongly encourage code deposition in a community repository (e.g. GitHub). See the Nature Portfolio guidelines for submitting code & software for further information.

Data

Policy information about availability of data

All manuscripts must include a data availability statement. This statement should provide the following information, where applicable:

- Accession codes, unique identifiers, or web links for publicly available datasets
- A description of any restrictions on data availability
- For clinical datasets or third party data, please ensure that the statement adheres to our policy

All data supporting the findings described in this manuscript are available in the article and in the Supplementary Information and Source Data, and from the corresponding author upon request. Data concerning TB burden, as well as countries demographic structures is available in open databases (WHO TB database: https://www.who.int/teams/global-tuberculosis-programme/data UN population division database: https://population.un.org/wpp/). The data concerning the M72/AS01E vaccine trial used here is publicly available at the original source (see main text references [9,10]). Source data are provided with this paper.

Human research participants

Policy information about studies involving human research participants and Sex and Gender in Research.

Reporting on sex and gender	N/A. This study does not involve field work. We analyze existing data which we do not disaggregate by sex and gender, but by age.
Population characteristics	In this study, we produced in-silico simulations of the multi-centric phase 2b clinical trial of the tuberculosis vaccine M72/AS01E, whose population characteristics are reported.
Recruitment	The simulated enrolment of participants in our set of simulated trials mimics the distribution of individuals in the original study across trial sites and age strata.
Ethics oversight	n/a

Note that full information on the approval of the study protocol must also be provided in the manuscript.

Field-specific reporting

Please select the one below that is the best fit for your research. If you are not sure, read the appropriate sections before making your selection.

☐ Life sciences ☐ Behavioural & social sciences ☒ Ecological, evolutionary & environmental sciences

For a reference copy of the document with all sections, see nature.com/documents/nr-reporting-summary-flat.pdf

Ecological, evolutionary & environmental sciences study design

All studies must disclose on these points even when the disclosure is negative.

Study description	The complex transmission chain of tuberculosis (TB) forces mathematical modelers to make mechanistic assumptions when producing prospective impact evaluations of novel vaccines at the population level. In this study, we produce in-silico simulations of clinical trials in order to ascertain the posterior probabilities of different model descriptions leaning on different vaccine mechanisms, given an efficacy estimate obtained from an actual clinical trial of vaccine efficacy, in our case, the multi-centric phase 2b clinical trial of the tuberculosis vaccine M72/AS01E. Next, we use these posteriors as Bayesian weights that measure the relative plausibility of each model given the observed efficacy in the trial under study. By doing so, we unlock the production of mechanism-agnostic impact forecasts for TB vaccines.
Research sample	Research sample is determined by the original M72/AS01E trial for the in-silico RCT simulations, where participants distributions across trial sites an d age groups were reported. In this study, the simulated enrolment of participants in our set of simulated trials mimics the distribution of individuals in the original study across trial sites and age strata.
Sampling strategy	The sampling strategy (i.e. distribution across sites and age groups) and sample size of our simulated trials (N=XX, and N=XX participants in the placebo and vaccine arms, respectively) needs to mimic the original M72/AS01E trial under analysis. Since these aspects are expected to impact the statistical power of our analyses, it is key to ensure that the simulated trials are based on the sampling strategy of the trial under study.
Data collection	Data was gathered by the authors either from public repositories (WHO TB database: https://www.who.int/teams/global-tuberculosis-programme/data UN population division database: https://population.un.org/wpp/) or from published studies, which are cited in the manuscript and in the data availability statement.
Timing and spatial scale	Data was not directly collected in the context of this study. The data used to model TB vaccines impact comes from static bibliographical sources (see Data Collection, above), as well as fom the WHO TB database: https://www.who.int/teams/global-

	tuberculosis-programme/data UN population division database: https://population.un.org/wpp/; which report TB burden and demographic data, respectively, at a yearly, and country-level resolution.
Data exclusions	No data was excluded from the analysis.
Reproducibility	We describe, and provide all the methods, software, data, and codes, used in this study to ensure reproducibility of the results.
Randomization	Random assignment of individuals of given age and trial sites, and initial status (F vs L), to placebo vs. vaccine cohorts, to mimic demographics of the recruited individuals in the M72/ASO1E trial is trivially achieved in our simulated trials by using stochastic random number generators built in R (version 3.6.3)
Blinding	Blinding was not required, as our work is based on in-silico simulated trials.

Did the study involve field work? ☐ Yes ☒ No

Reporting for specific materials, systems and methods

We require information from authors about some types of materials, experimental systems and methods used in many studies. Here, indicate whether each material, system or method listed is relevant to your study. If you are not sure if a list item applies to your research, read the appropriate section before selecting a response.

Materials & experimental systems

n/a	Involved in the study
☒	☐ Antibodies
☒	☐ Eukaryotic cell lines
☒	☐ Palaeontology and archaeology
☒	☐ Animals and other organisms
☒	☐ Clinical data
☒	☐ Dual use research of concern

Methods

n/a	Involved in the study
☒	☐ ChIP-seq
☒	☐ Flow cytometry
☒	☐ MRI-based neuroimaging
